# Effect of Ankle-Foot Orthosis on Paretic Gastrocnemius and Tibialis Anterior Muscle Contraction of Stroke Survivors During Walking: A Pilot Study

**DOI:** 10.3390/bios14120595

**Published:** 2024-12-04

**Authors:** Wei Liu, Hui-Dong Wu, Yu-Ying Li, Ringo Tang-Long Zhu, Yu-Yan Luo, Yan To Ling, Li-Ke Wang, Jian-Fa Wang, Yong-Ping Zheng, Christina Zong-Hao Ma

**Affiliations:** 1 Department of Biomedical Engineering, The Hong Kong Polytechnic University, Hong Kong SAR, China; 21118892r@connect.polyu.hk (W.L.); ringo-tanglong.zhu@connect.polyu.hk (R.T.-L.Z.); 23054595r@connect.polyu.hk (Y.-Y.L.); jane.yt.ling@connect.polyu.hk (Y.T.L.); likewang@eieling.com (L.-K.W.); yongping.zheng@polyu.edu.hk (Y.-P.Z.); 2 Department of Prosthetic and Orthotic Engineering, School of Rehabilitation, Kunming Medical University, Kunming 650500, China; 16903232r@connect.polyu.hk (H.-D.W.); 20230312@kmmu.edu.cn (Y.-Y.L.); 3 Research Institute for Smart Ageing, The Hong Kong Polytechnic University, Hong Kong SAR, China; 4 Centre for Developmental Neurobiology, Institute of Psychiatry, Psychology and Neuroscience, King’s College London, London WC2R 2LS, UK; 5 Beijing Sonoptek Co., Ltd., Beijing 100048, China; wangjf@sonoptek.com

**Keywords:** Paretic muscle, ankle muscle, muscle morphology, muscle structure, ultrasound imaging, gait, post-stroke

## Abstract

Ankle-foot orthoses (AFOs) have been commonly prescribed for stroke survivors with foot drop, but their impact on the contractions of paretic tibialis anterior (TA) and medial gastrocnemius (MG) has remained inconclusive. This study thus investigated the effect of AFOs on these muscle contractions in stroke survivors. The contractions of paretic TA and MG muscles were assessed in twenty stroke patients and compared between walking with and without AFOs, using a novel wearable dynamic ultrasound imaging and sensing system. The study found an increase in TA muscle thickness throughout a gait cycle (*p* > 0.05) and a significant increase in TA muscle surface mechanomyography (sMMG) signals during the pre- and initial swing phases (*p* < 0.05) when using an AFO. MG muscle thickness generally decreased with the AFO (*p* > 0.05), aligning more closely with trends seen in healthy adults. The MG surface electromyography (sEMG) signal significantly decreased during the initial and mid-swing phases when wearing an AFO (*p* < 0.05). The TA-MG co-contraction index significantly decreased during initial and mid-swing phases with the AFO (*p* < 0.05). These results suggest that AFOs positively influenced the contraction patterns of paretic ankle muscles during walking in stroke patients, but further research is needed to understand their long-term effects.

## 1. Introduction

Foot drop is a prevalent and debilitating condition that often appears following a stroke. It is characterized by weakened or absent voluntary ankle dorsiflexion and a dragging or slapping gait pattern. This issue can result in diminished balance and poor mobility [1], as well as an elevated risk of falling [2]. As a result, individuals who have had a stroke may experience restrictions in their daily activities [3] and a lower quality of life [4]. The primary cause of foot drop after a stroke is typically associated with muscle weakness or paralysis in the ankle dorsiflexors, often accompanied by high muscle tone and possible contracture in ankle plantar flexors [5].

There are various treatment options available to manage foot drop and improve functional outcomes following a stroke. Physical therapy plays a crucial role in rehabilitation, focusing on strengthening the affected/paretic muscles, improving balance and coordination, and retraining the affected muscles to regain control and function [6,7]. Besides physical therapies, certain assistive devices, such as an ankle-foot orthosis (AFO), can provide stability and prevent foot drop by maintaining the foot in a neutral or slightly dorsiflexed position [8]. This helps to enhance foot clearance while walking and reduces the risk of falls in stroke patients [8].

Understanding more on the changes in paretic ankle muscle activity while wearing AFOs could offer more evidence for future clinical practice. Previous research has demonstrated that AFOs can promptly enhance an individual’s walking speed, balance, energy expenditure, and overall gait biomechanics [9,10]. However, how AFOs altered the muscle activity of dorsiflexors and plantar flexors of the paretic side to achieve such improved balance and gait performance in post-stroke patients has remained unconcluded. Most previous studies have used surface electromyography (sEMG) to study the changes in stroke individual’s ankle muscle electrical activity with and without wearing an AFO. Murayama and Yamamoto used sEMG to monitor tibialis anterior (TA) and soleus (SOL) muscles’ electrical activity during walking [11]. They found that the plantarflexion movement reduced by AFOs can augment the activity of the TA muscle during the loading response phase and the activity of the SOL muscle during the stance phase, resulting in improved stability and propulsion. Meanwhile, traditional treatment approaches may sometimes avoid the use of AFOs due to concerns about possible disuse atrophy of the TA muscle [12,13], as immobilization by AFO could inhibit the eccentric contraction of the TA muscle during walking. Some previous studies have indicated that individuals using AFOs exhibited reduced sEMG activity of the TA muscle from the swing to the loading response phase, as compared to those walking without an AFO [12,13]. Similarly, Ohata et al. reported reduced sEMG amplitude in the gastrocnemius muscle when stroke patients wore AFOs [14]. This suggests a risk of disuse atrophy with prolonged AFO use. In contrast, Mulroy et al. have found no significant difference in the sEMG activity of TA and SOL muscles in stroke patients walking with an AFO compared to without it [15]. Nikamp et al. have observed a marked reduction in TA muscle sEMG activity among individuals using AFOs during the initial treatment phase; however, this difference diminished after 26 weeks of use [16]. Similar findings were also reported in the study conducted by Geboers et al. [17]. Therefore, it is essential to further explore the impact of using AFOs on paretic ankle dorsiflexors and plantar flexors in stroke survivors, especially with the potential application of more advanced technologies.

A variety of sensors and tools have been employed to study and assess muscle activity in different conditions. Among them, sEMG detects muscle electrical signals that transmitted from the peripheral nervous system, while surface mechanomyography (sMMG) measures muscle mechanical activities from the skin surface after receiving the electrical signal [16,17,18,19,20]. This has made the sMMG more resistant to interference compared to sEMG. Combining both techniques could achieve a more comprehensive and accurate assessment of muscle electrical and mechanical activity in complex environments, and thus enhance the understanding of muscle performance and adaptations during various treatments. Some imaging techniques, such as magnetic resonance imaging (MRI), could visualize muscle structure and morphology, further advancing the assessment of post-stroke muscle atrophy by quantify related parameters (i.e., muscle volume and cross-sectional area [21]). However, the application of MRI is limited by high costs, long time consumption, and challenges in dynamic settings [22].

Ultrasound imaging offers a non-invasive and affordable alternative for assessing muscle morphology without ionizing radiation in static conditions [23]. Studies by Monjo et al. [24] and Gonzalez-Buonomo et al. [25] have shown reduced muscle thickness in the paretic lower extremity of post-stroke individuals using ultrasound imaging. However, the previous technical constraints have restricted these technologies to static evaluations (i.e., recumbent [24,25] or sitting [24] position) only, making it difficult to evaluate the changes in muscle morphology and contraction pattern in dynamic situations.

Assessing muscle activity and morphology in dynamic activities (e.g., walking) is crucial for understanding patients’ recovery and mobility in post-stroke patients. It provides insights into muscle efficiency, coordination, and compensatory strategies, facilitating the prescription and adjustment of individualized rehabilitation programs to improve patient’s gait and quality of life. However, most of the previous muscle ultrasound imaging devices have been cumbersome, limiting their applications into static assessment of muscle volume (product of muscle length and physiological cross-sectional area) [24,25] and muscle stiffness [26] when the muscle is at rest. Further optimizations of the conventional large ultrasound imaging devices are needed to make them more portable and mobile.

To understand lower-extremity muscle activity and morphology during dynamic conditions (e.g., walking), a wearable ultrasound imaging and sensing system has been developed and validated by our team [20,27]. This system consisted of a wearable ultrasound probe and multiple motion sensors to simultaneously measure the muscle ultrasound image, sEMG, and sMMG, as well as the plantar force underneath the foot, during standing and walking [27]. It has been applied in healthy young adults and post-stroke individuals to capture their ankle muscle activity during natural walking without any interventions [20,27]. However, changes in ankle muscle activity in post-stroke patients while walking with AFOs has remained unclear and warranted further study.

Therefore, this study utilized the wearable ultrasound imaging and sensing system to examine the impact of wearing a solid AFO on the contraction patterns and morphological variations of TA and medial gastrocnemius (MG) muscles in stroke survivors during walking. It was hypothesized that the use of AFOs would positively influence the contraction patterns of these muscles on the paretic side post-stroke. The findings would provide valuable insights for the orthotic management of foot drop in stroke survivors and enhance evidence-based practice.

## 2. Materials and Methods

### 2.1. Participants

Participants should meet the following inclusion criteria: (1) the initial stroke episode was within 6 months prior to the study; (2) diagnosed with hemiparesis due to unilateral ischemic or hemorrhagic stroke; (3) with a Brunnstrom Scale score ranging from III to VI; (4) capable of walking for at least eight meters without assistance; and (5) with passive ankle dorsiflexion range of motion of at least 0° (i.e., the neutral position). Patients were excluded if they had any (1) cognitive impairment, (2) uncontrolled cardiovascular or respiratory disorders, or (3) fracture or muscle disorders affecting mobility. Sub-acute stroke survivors were recruited because the sub-acute phase is a critical period for rehabilitation. During this phase, patients commonly exhibit the highest potential for motor recovery, making it an optimal time for receiving various interventions. Informed consent forms were signed once the eligible patients agreed to participate in this study. This study was registered in the International Clinical Trials Registry Platform (ICTRP) via the Chinese Clinical Trial Registry (Ref: ChiCTR2300074539). Ethical approval was granted by the Kunming Medical University Medical Ethics Committee (Ref: KMMU2023MEC149), and all procedures were conducted in accordance with the Helsinki Declaration of 1975.

### 2.2. Wearable Ultrasound Imaging and Sensing System

**Figure 1:**
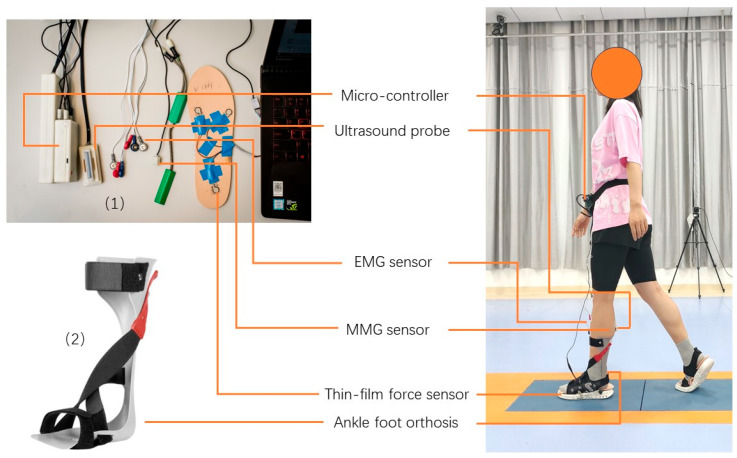
Illustration of a participant using: (1) the wearable ultrasound imaging and sensing system and (2) the solid posterior leaf-spring ankle foot orthosis (AFO) during walking.

As shown in Figure 1, the wearable system included a wearable ultrasound probe (band width: 7.5 MHz ± 35%, frame rate: 10 Hz), two sets of sEMG electrodes (272-Bx, Noraxon USA Inc., Scottsdale, AZ, USA), an sMMG sensor (N1000060, VTI Technologies Oy, Vantaa, Finland), and three thin-film force sensors (A301, Tekscan Co., Ltd., South Boston, MA, USA). These components were used to capture the muscle’s real-time B-mode ultrasound image, electrical activity, mechanical activity, and the plantar force for identifying gait cycles, respectively [27].

### 2.3. Experimental Procedure

Subjective assessments were first conducted for each participant. A comprehensive description of the experimental procedures was given. The participant’s stage of stroke recovery was assessed using the Brunnstrom Approach [28], muscle tone of plantar flexors at the paretic side was assessed using the Modified Ashworth Scale [29], and balance performance was assessed using the Berg Balance Scale [30] in accordance with the established protocols [31].

Each participant completed instrumented walking trials with the wearable ultrasound imaging and sensing system placed on the paretic side. Before the trials, the ultrasound images of the paretic MG and TA muscles were captured separately for each participant in a comfortable standing position. These images served as the baseline muscle thickness measurements for the study. The participant was then fitted with a prefabricated solid posterior leaf-spring AFO (272-Bx, Noraxon USA Inc., Scottsdale, AZ, USA), following a certified orthotist’s instructions. The AFO, available in various sizes, was worn within a sandal, as illustrated in Figure 1. The participant was directed to walk for approximately twenty meters to acclimate to the AFO, during which they were instructed to actively activate/contract the paretic TA and MG muscles while walking with the AFO. Subsequently, the participant was equipped with the wearable ultrasound imaging and sensing system. The ultrasound probe was positioned longitudinally on the paretic MG muscle belly, with one set of sEMG electrodes and the sMMG sensor placed parallel to the ultrasound probe on the paretic MG muscle belly. Another set of sEMG electrodes was positioned on the paretic TA muscle. Additionally, three thin-film force sensors were placed underneath the first and fifth metatarsal heads (1st and 5th MTH) and the heel of the paretic foot. Once properly set up, the participant was instructed to walk three consecutive trials on flat ground at a comfortable speed, with each trial covering approximately eight meters. The same procedures were repeated to evaluate the paretic MG muscle activity while walking without an AFO, and the paretic TA muscle activity while walking with and without an AFO [20]. The sequence for capturing TA and MG muscle activity with and without wearing the AFO was randomized for each participant.

### 2.4. Data Processing and Analysis

The gait cycle was identified according to the measurements of pressure sensors underneath the plantar foot. For each walking condition, three complete gait cycles were extracted from the middle portion of each of the three walking trials for further processing using MATLAB (Version 2016b, The MathWorks Inc., Natick, MA, USA). To ensure consistency, all data were resampled to represent the 0–100% gait cycle with a 5% interval [27]. Each gait cycle was divided into seven phases: loading response (LR, 0–10%), mid-stance (MSt, 10–30%), terminal stance (TSt, 30–50%), pre-swing (PSw, 50–60%), initial swing (ISw, 60–70%), mid-swing (MSw, 70–85%), and terminal swing (TSw, 85–100%) phases, by referring to a previous publications on gait analysis of stroke survivors [18,32].

### 2.5. sMMG and sEMG Signal Processing and Analysis

The sMMG and sEMG data were filtered using a fourth-order Butterworth band-pass filter (5–50 Hz for MMG and 30–500 Hz for EMG), followed by rectification and additional filtering with a moving-average filter using a temporal window of 0.101 s. The data were then normalized to the peak values that were observed within the three extracted gait cycles [27]. The mean amplitude value of the processed signal within each 5% interval of a gait cycle was used for further statistical analysis.

Additionally, regarding the sEMG data, the participant’s co-contraction index (CI) was calculated and examined to evaluate the co-contraction pattern of TA and MG muscles in various gait phases while walking. In the LR, ISw, MSw, and TSw phases, the TA muscle functioned as the agonist muscle, whereas the MG muscle functioned as the antagonist muscle. The CI was thereby calculated as [18]:

(1) $$CI=\frac{2I_{MG}}{I_{MG}+I_{TA}}\times 100$$

In the MSt and TSt phases, the MG muscle was anticipated to be the agonist muscle, while the TA muscle was expected to be the antagonist muscle. The CI was thereby calculated as [18]:

(2) $$CI=\frac{2I_{TA}}{I_{MG}+I_{TA}}\times 100$$

As detailed in the study by Banks, et al. [18], sEMG is represented on the longitudinal axis, while the 100% gait cycle is depicted on the horizontal axis. In Equations (1) and (2), I_MG_ was determined by calculating the integral area under the curve of the normalized sEMG signals from the MG muscle. Similarly, I_TA_ was calculated as the integral area under the curve of the normalized sEMG signals from the TA muscle, during the corresponding phases. The CI was calculated for the respective phases. The CI was computed for each gait phase, excluding the PSw phase, due to the reversal of roles between the TA and MG muscles as agonist and antagonist during this phase [18].

### 2.6. Ultrasound Image Processing and Analyses

A skilled practitioner manually annotated all the muscle ultrasound imaging data by marking the upper and lower muscle boundaries on each extracted ultrasound imaging frame post-experiment. A custom MATLAB (Version 2016b, The MathWorks Inc., Natick, MA, USA) algorithm was used to determine the muscle area by measuring the area between the marked upper and lower muscle boundaries on each ultrasound imaging frame. The muscle area was then divided by the width of the ultrasound image (30 mm) to calculate the average muscle thickness for each ultrasound image. The average muscle thickness values from three consecutive baseline ultrasound images, captured while the participant’s ankle joint was in a comfortable standing position, were averaged to establish the baseline muscle thickness. This baseline thickness was considered the reference value and set as “100%” during the normalization process. To normalize the ultrasound imaging data, the average muscle thickness of each ultrasound imaging frame was divided by the baseline muscle thickness first and then multiplied by 100%, resulting in the normalized average muscle thickness in a percentage. The mean of such normalized thickness values within each 5% interval of a gait cycle was used for further statistical analyses [20].

### 2.7. Statistical Analyses

Statistical analyses were conducted using SPSS 25.0 software, with a significance level set at 0.05. The Shapiro-Wilk test was used for testing the normality of the data. The test-retest reliability of the collected data across three repetitive gait cycles for each walking condition was assessed using the intraclass correlation coefficient (ICC). The paired *t*-test was used to compare the differences in normalized muscle thickness, sEMG, sMMG, and plantar force of the paretic side among participants, with and without the use of an AFO during walking.

## 3. Results

### 3.1. Demographics and Subjective Assessment Results

As shown in Table 1, a total of 20 sub-acute stroke survivors (13 males and 7 females) were recruited from three hospitals in Yunnan province, China. The average age of the participants was 53.0 ± 13.3 years (mean ± standard deviation), and their average body mass index (BMI) was 24.5 ± 2.7. All participants were within 1 to 6 months post-stroke, with Brunnstrom Scale scores ranging from III to VI. The muscle tone of the plantar flexors, as assessed by the Modified Ashworth Scale, was 1.6 ± 1.3, and the Berg Balance Scale Score was 35.6 ± 8.8.

### 3.2. Test–Retest Reliability

Table 2 displays the ICC values for the various parameters, including muscle thickness, sEMG signal, sMMG signal, and plantar force within each 5% interval of a gait cycle for different walking conditions. The test-retest reliability of the wearable system for measuring TA and MG muscle thickness remained consistently excellent among participants, regardless of AFO usage, with ICC values ranging from 0.880 to 0.991 (TA) and 0.876 to 0.994 (MG), respectively (*p* < 0.05). For the sEMG signals of the MG and TA muscles, most ICC values exceeded 0.70, with a small portion ranging from 0.46 to 0.70 (*p* < 0.05). The ICC values for the sMMG signals of the TA and MG muscles were relatively lower and more variable, with most values falling between 0.61 and 0.88, and a few between 0.39 and 0.59 (*p* < 0.05). The ICC values for the wearable system’s repeated measurements of plantar pressure during walking ranged from 0.62 to 0.89 (*p* < 0.05).

### 3.3. Muscle Thickness

**Figure 2:**
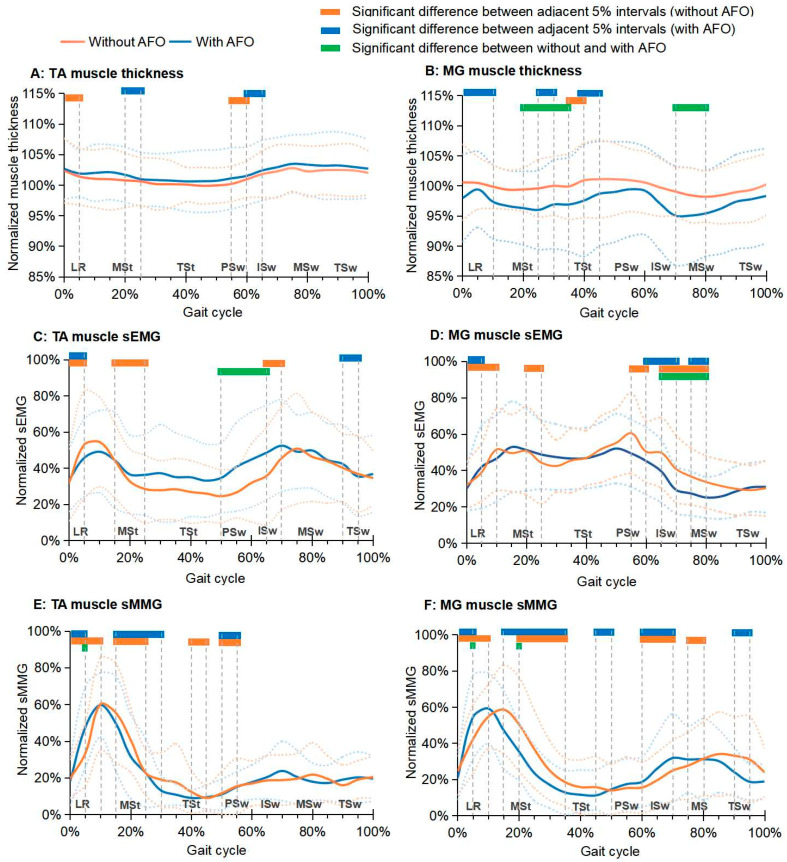
Changes in average normalized thickness, sEMG, and sMMG of the TA and MG muscles of the paretic side during a gait cycle, while the participants walked without and with an AFO. (Note: the solid lines and dashed lines represent the mean and mean ± standard deviation of each average measurement, respectively; TA, tibialis anterior muscle; MG, medial head of gastrocnemius muscle; sEMG, surface electromyography; sMMG, surface mechanomyography; LR, loading response; MSt, mid-stance; TSt, terminal stance; PSw, pre-swing; ISw, initial swing, MSw, mid-swing; TSw, terminal swing).

Figure 2A,B illustrate the average normalized thickness of the TA and MG muscles at the paretic side of participants during walking, both with and without the use of an AFO. When wearing the AFO at the paretic side, the normalized TA muscle thickness significantly decreased during the MSt phase (20–25% of a gait cycle) and significantly increased during the ISw phase (60–65% of a gait cycle) (*p* < 0.05). Conversely, when not wearing the AFO at the paretic side, the normalized TA muscle thickness significantly decreased during the LR phase (0–5% of a gait cycle) and significantly increased during the PSw phase (55–60% of a gait cycle) (*p* < 0.05). Additionally, the normalized MG muscle thickness demonstrated significant changes during the LR, MSt, and TSt phases (0–10%, 25–30%, and 40–45% of a gait cycle, respectively) while wearing the AFO (*p* < 0.05), and only showed a significant increase during the TSt phase (35–40% of a gait cycle) when not wearing the AFO (*p* < 0.05).

In the comparison of walking with and without AFO at the paretic side, the normalized TA muscle thickness appeared to be greater when wearing the AFO than that of without wearing it throughout a gait cycle; however, this difference was not statistically significant (*p* > 0.05, Figure 2A,B). In contrast, a significantly and consistently smaller normalized MG muscle thickness was observed when wearing the AFO comparing to not wearing it, with a significant 2.8–4.0% reduction primarily observed during the MSt, TSt, and MSw phases (20–35% and 70–80% of a gait cycle, *p* < 0.05).

### 3.4. Muscle Electrical and Mechanical Activity

Figure 2C–F illustrate the average normalized sEMG and sMMG signals of the TA and MG muscles at the paretic side during walking in participants, both without and with the use of an AFO. In relation to the TA muscle, a significant 10.1–14.1% increase in the normalized sEMG signal was observed during the PSw and ISw phases (50–65% of a cycle gait) when the AFO was used (*p* < 0.05). Additionally, a significant 14.2% increase in the normalized sMMG signal was noted during the LR phase (5% of a gait cycle) when the participants were walking with an AFO (*p* < 0.05). Concerning the MG muscle, a significant 8.5–11.5% decrease in the normalized sEMG signal was observed during the ISw and MSw phases (65–80% of a gait cycle) with AFO use (*p* < 0.05). The normalized sMMG signal showed a significant increase (12.3%) during the LR phase (5% of a gait cycle) and a reduction (15.1%) during the MSt phase (20% of a gait cycle) when using the AFO (*p* < 0.05).

**Figure 3:**
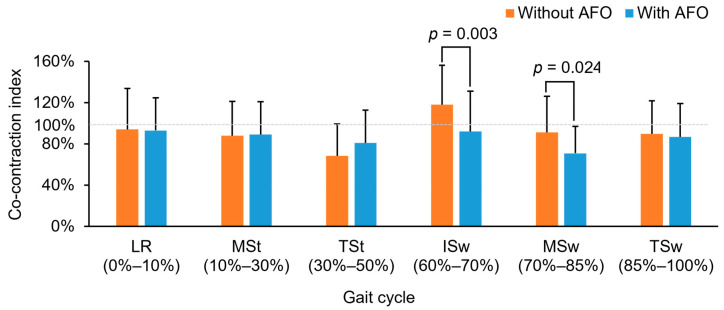
Co-contraction index (CI) of the TA and MG muscles of the paretic side during different gait phases while participating stroke survivors walked without and with an AFO. (Note: AFO, ankle-foot orthosis; LR, loading response; MSt, mid-stance; TSt, terminal stance; Isw, initial swing, MSw, mid-swing; TSw, terminal swing; dotted line: 100% of co-contraction index).

Figure 3 presents the CI values of the TA and MG muscles at the paretic side in participating stroke survivors, both without and with the use of an AFO, across various gait phases. When walking without the AFO, the CI ranged from 68% to 118%; with AFO, it ranged from 71% to 93% throughout the gait cycle of the participants. The fluctuation decreased from 50% to 22% after the fittings of AFOs. Additionally, significant difference in CI were observed between the MSt and TSt phases (*p* < 0.05), TSt and ISw phases (*p* < 0.05), and ISw and MSw phases (*p* < 0.001) when walking without an AFO. However, significant differences were only observed between the ISw and MSw phases (*p* < 0.05), and MSw and TSw phases (*p* < 0.05), when walking with an AFO. Moreover, there were significant reductions (20.4% and 26.1%) in the CI during the ISw and MSw phases when walking with the AFO compared to walking without the AFO (*p* < 0.05).

### 3.5. Plantar Force

**Figure 4:**
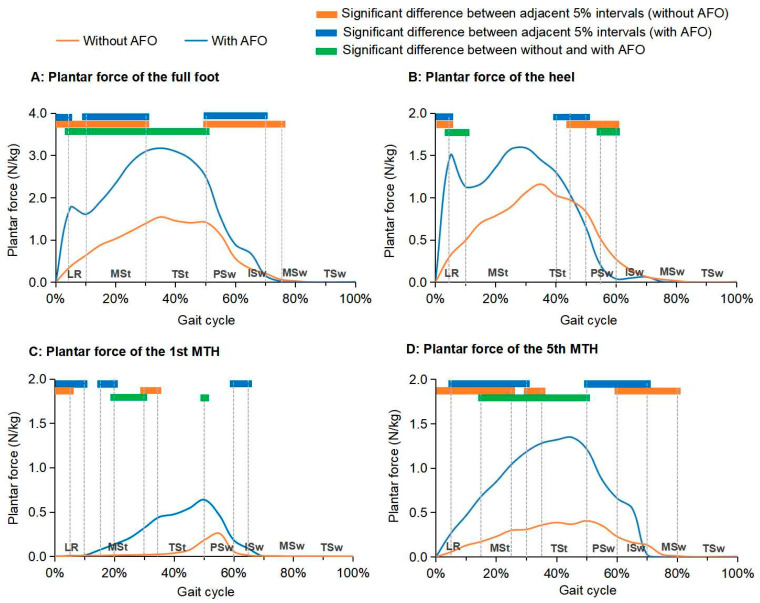
Plantar force of the full foot, heel, 1st MTH, and 5th MTH of participating stroke survivors while walking without and with an AFO (n = 20). (Note: AFO, ankle-foot orthosis; MTH, metatarsal head; LR, loading response; MSt, mid-stance; TSt, terminal stance; PSw, pre-swing; Isw, initial swing, MSw, mid-swing; TSw, terminal swing).

Figure 4A–D depict the distribution of plantar force across the full foot, heel, 1st MTH, and 5th MTH at the paretic side of the participants during the stance phase, while walking both without and with the AFO. A significant increase (1.0–1.7 N/kg) in the plantar force of the full foot was noted throughout most of the stance phase (5–50% of a gait cycle), when walking with the AFO comparing to without it (*p* < 0.05). In terms of the heel, the plantar force significantly increased (0.6–1.2 N/kg) during the LR phase (5–10% of a gait cycle), but significantly decreased (0.2–0.3 N/kg) during the PSw phase (55–60% of a gait cycle) when using the AFO compared to not using it (*p* < 0.05). Additionally, when an AFO was worn, a significant increase in plantar force was also found at the 1st MTH during the MSt and TSt phases (20–30% and 45–50% of a gait cycle) and the 5th MTH during the MSt and TSt phases (15–50% of a gait cycle), with an increase of 0.1–0.5 N/kg and 0.5–1.0 N/kg, respectively (*p* < 0.05).

### 3.6. Temporal Gait Parameters

Table 3 displays the alterations of temporal gait parameters at the paretic side while participants walked with and without an AFO. When walking with an AFO, the participants exhibited a significant decrease in stride time, stance time, and swing time, compared to walking without an AFO (*p* < 0.001). Additionally, the proportion of the stance phase in a gait cycle significantly decreased, while the proportion of the swing phase significantly increased in participants while walking with an AFO (*p* < 0.05).

## 4. Discussion

This study has investigated the effect of wearing solid AFO on the paretic TA and MG muscle contraction patterns during different gait phases in sub-acute stroke survivors by using an innovative wearable ultrasound imaging and sensing system to thoroughly examine the internal activity and morphology of the TA and MG muscles in stroke survivors while walking with and without an AFO on the paretic side. The study results generally supported the hypothesis that solid AFO usage could positively affect the contraction patterns of paretic TA and MG muscles, as well as gait asymmetry and weight bearing, during walking in post-stroke patients. More detailed discussions can be found below.

### 4.1. Test-Retest Reliability

The wearable ultrasound imaging and sensing system has demonstrated good to excellent test-retest reliability in capturing the thickness, sEMG signal, and sMMG signal of TA and MG muscles, as well as the plantar pressure on the paretic side during walking, in stroke survivors both with and without an AFO. The ICC values have been generally comparable to those reported for healthy people [27] and stroke survivors [20].

### 4.2. Muscle Thickness

A consistently slight increase was observed in paretic TA muscle thickness when an AFO was used during walking in the participating stroke survivors. This increase may be contributed by the passively dorsiflexed ankle joint that maintained by the solid AFO in stroke survivors, as compared to the original foot drop position (or plantarflexed ankle joint) while they were walking without an AFO. Correspondingly and conversely, this study did observe a significant reduction in the MG muscle thickness while participants were walking with an AFO. This reduction could be due to the passive elongation of the MG muscle upon passively dorsiflexing the paretic ankle joint with an AFO, resulting in decreased MG muscle thickness. Furthermore, the fluctuations in the MG muscle thickness throughout the gait cycle were more pronounced with the use of an AFO compared to walking without an AFO. The observed trend in muscle thickness variation with AFO application appears to align more closely with the patterns, particularly during the ISw and MSw phases, documented in healthy young adults by Ma et al. [27], yet contrasting with the patterns observed during walking without an AFO. These results indicate that the use of AFOs may facilitate a more natural or normal contraction of the paretic MG muscle during walking for stroke survivors. Further research could involve the development and application of wearable ultrasound imaging systems with enhanced imaging quality, which could enable the visualization of muscle fascicle length and pennation angle, to further validate these observations.

### 4.3. Muscle Electrical Activity

This study has observed the generally similar paretic TA sEMG trend during walking in stroke patients, regardless of walking with or without an AFO, as that of a previous study reporting stroke survivors’ muscle activity during natural walking [20]. In this study, the increased paretic TA sEMG signal in the stance phase while using AFOs may suggest that the AFO could facilitate TA muscle contraction. This study has also observed that the use of AFOs could significantly increase TA muscle activity during the ISw phase. This contradicts the previously reported finding that using AFOs could decline TA muscle contraction during the swing phase, since the AFO could help maintain ankle joint in a neutral position, which may suppress users from actively contracting the TA muscles by themselves [13,16]. This inconsistency may be attributed to the walking or gait training instructions that the participants underwent with the AFO prior to the walking trials in this study, in which all participants were guided to engage the TA and MG muscles while walking with the AFO by an experienced physiotherapist. This suggests that appropriate walking training with an AFO for stroke patients may help to stimulate TA muscle activity during walking. This finding warrants further investigation and verification in the long term.

Aligning with the decreased thickness of the MG muscle, this study also observed significant reductions in MG sEMG signals during the Isw and MSw phases, while participants walked with an AFO compared to without an AFO. This study also observed that MG muscle thickness significantly decreased during the MSw phase, while MG sEMG signals significantly decreased during the Isw and MSw phases. This may be explained by delays in MG muscle activity where the timing of muscle sEMG signal generation was commonly earlier than that of muscle internal morphological contraction [33]. It is also noteworthy to observe significant differences in MG muscle thickness between the two conditions (with and without the AFO) during the MSt and TSt phases based on ultrasound images. However, no significant difference was found in the MG sEMG signal during these two phases. Similarly, Choo et al. has used ultrasound imaging to detect the oropharyngeal swallowing events, and demonstrated its further ability to capture the tongue base retraction and hyolaryngeal excursion during swallowing more accurately in young adults, which could not be detected using the sEMG electrodes [34]. These results imply that ultrasound imaging could perform more effectively when capturing the internal morphological changes in muscles during dynamic activities; the sEMG has been limited to detecting the muscle electrical activity from the skin surface, which may introduce more noise and crosstalk among neighboring muscles. This supports the potential benefits of integrating both ultrasound imaging and sEMG measurements in future studies, to gain a more comprehensive understanding of lower-limb muscle activity across different dynamic conditions in stroke survivors.

The significantly smaller CI values observed in the paretic TA and MG muscles during the Isw and MSw phases suggest an improved co-contraction pattern between the antagonist and agonist ankle muscles. This improved co-contraction may facilitate better ankle control and reduced energy expenditure during the ISw and MSw phases in sub-acute stroke patients while walking with an AFO, as compared to walking without an AFO [18]. The reduced CI values may be contributed to by the more natural contraction of the paretic MG muscle during these two phases when using the AFO, as supported by the documented reduced thickness of the paretic MG muscle in this study. Additionally, the overall reduced variability in CI values (i.e., reduced from 50% to 22%) observed throughout a gait cycle may also indicate a more stable co-contraction of the TA and MG muscles when using an AFO. This increased stability in co-contraction may also contribute to a reduction in energy consumption during walking. Nevertheless, further research is needed to confirm these intriguing findings.

### 4.4. Plantar Force and Gait Cycle

By controlling the abnormal foot drop and plantar flexion using an AFO at the paretic side, the weight-bearing capacity of the paretic side during stance phase has significantly increased in participating stroke survivors in this study. Additionally, it has also allowed the paretic ankle joint to adapt and accept loading more quickly during the loading response phase, and thereby reducing the time spent in the stance phase in stroke survivors. Consequently, the proportions of the stance and swing phases in the gait cycle has improved, leading to a more symmetrical gait pattern and increased walking speed in stroke survivors using an AFO. Such findings of this study have also been in accordance with the findings reported in previous studies [12,35].

### 4.5. Limitations

Several limitations should be acknowledged when interpreting the findings of this pilot study. Firstly, this study did not collect data from the nonparetic side to compare between the paretic and nonparetic sides. This makes it difficult to assess how using an AFO on the paretic side would affect the nonparetic side’s performance during walking in stroke survivors. Given the impaired function of the paretic side, the nonparetic side may compensate during walking, as reported in previous studies [36]. Further studies are needed to investigate and compare the difference between the two sides. Secondly, this study only examined the immediate impact of AFO use on the thickness and activity of the TA and MG muscles. Future research should explore the long-term effects to have more generalized findings. Thirdly, while this study has taken several steps to minimize the electrical crosstalk between agonist (e.g., medial gastrocnemius) and nearby antagonist (e.g., tibialis anterior) muscles, crosstalk may still exist among different muscles. This may have confounded the reported results in the sEMG and muscle co-contraction index (CI). Fourthly, there was no force sensor on the great toe and only three force sensors were put on the plantar surface of the foot, including the first and fifth metatarsal heads and the heel. While the pilot study has demonstrated that the stroke survivors walking with a solid ankle-foot orthosis (AFO) did not exhibit dorsiflexion of the metatarsophalangeal joints at the paretic side, it is still possible that the lack of the force sensor on the big toe may lead to a shortened stance phase time at the paretic side, as measured by the wearable ultrasound imaging and sensing system. Future studies shall consider adding or placing a force sensor underneath the big toe to have a more appropriate extraction of the stance phrase. Finally, it should be cautious when applying the findings of the current study to stroke patients in other recovery phases, as this study only focused on sub-acute stroke patients. Sub-acute stroke patients tend to exhibit the highest potential for motor recovery; therefore other patients may have different responses and changes in motor recovery.

## 5. Conclusions

The results of this pilot study generally supported that the use of AFOs could positively affect the contraction patterns of paretic TA and MG muscles, as well as weight-bearing capacity and gait symmetry, during walking for sub-acute stroke survivors. However, future research is still needed to understand the long-term effects of AFO use in stroke patients.

## Figures and Tables

**Table 1:** Characteristics of participating stroke survivors (n = 20).

Gender (M/F)	Cause of Stroke (Hemorrhagic/Ischemic)	Age (Years)	BMI	Brannstrom Scale (III/IV/V/VI)	Months Since Stroke	Paretic Leg (L/R)	Modified Ashworth Scale	Berg Balance Scale
13/7	8/12	53.0 ± 13.3 (range: 39.0–68.0)	24.5 ± 2.7 (range: 18.7–29.3)	9/8/2/1	2.9 ± 2.0 (range: 1.0–6.0)	9/11	1.6 ± 1.3 (range: 0–4)	35.6 ± 8.8 (range: 29.0–53.0)

(Note: M/F: male/female; BMI: body mass index; L/R: left/right; Modified Ashworth Scale: the tone of the gastrocnemius muscle on the paretic side; data are presented as mean ± standard deviation and range).

**Table 2:** ICC values of the repeated measurements obtained from the wearable ultrasound imaging and sensing system during a gait cycle of the participating stroke survivors without and with AFOs.

Gait Cycle	Paretic Side Without AFO							Paretic Side with AFO						
	Thickness _MG	Thickness _TA	sMMG _MG	sMMG _TA	sEMG _MG	sEMG _TA	Plantar Force	Thickness _MG	Thickness _TA	sMMG _MG	sMMG _TA	sEMG _MG	sEMG _TA	Plantar Force
0%	0.988	0.936	0.881	0.855	0.791	0.900	NA	0.951	0.989	0.637	0.810	0.716	0.657	NA
5%	0.974	0.950	0.677	0.643	0.706	0.803	0.816	0.935	0.991	0.651	0.806	0.771	0.704	0.843
10%	0.966	0.952	0.668	0.546	0.797	0.859	0.859	0.912	0.979	0.632	0.794	0.707	0.689	0.777
15%	0.950	0.931	0.771	0.621	0.759	0.710	0.888	0.901	0.984	0.746	0.828	0.68	0.833	0.770
20%	0.965	0.930	0.715	0.819	0.760	0.791	0.786	0.934	0.981	0.574	0.732	0.774	0.742	0.721
25%	0.956	0.944	0.525	0.632	0.795	0.679	0.814	0.925	0.980	0.615	0.619	0.824	0.880	0.762
30%	0.968	0.959	0.658	0.541	0.856	0.891	0.736	0.917	0.981	0.650	0.685	0.974	0.893	0.694
35%	0.97	0.956	0.733	0.661	0.938	0.652	0.662	0.921	0.985	0.391	0.629	0.638	0.860	0.742
40%	0.977	0.943	0.656	0.490	0.723	0.797	0.763	0.932	0.986	0.690	0.411	0.724	0.843	0.790
45%	0.975	0.957	0.523	0.582	0.741	0.693	0.854	0.912	0.986	0.591	0.604	0.820	0.878	0.794
50%	0.973	0.943	0.509	0.646	0.785	0.652	0.842	0.970	0.987	0.582	0.787	0.876	0.870	0.636
55%	0.975	0.948	0.642	0.568	0.699	0.440	0.630	0.919	0.986	0.653	0.771	0.860	0.947	0.623
60%	0.994	0.922	0.561	0.681	0.816	0.583	0.609	0.900	0.980	0.638	0.902	0.653	0.955	0.805
65%	0.967	0.899	0.520	0.688	0.827	0.702	0.623	0.880	0.988	0.751	0.816	0.620	0.871	0.718
70%	0.962	0.877	0.726	0.688	0.783	0.643	NA	0.957	0.986	0.811	0.814	0.771	0.752	NA
75%	0.957	0.876	0.794	0.613	0.802	0.628	NA	0.968	0.988	0.681	0.692	0.469	0.553	NA
80%	0.945	0.893	0.640	0.512	0.815	0.663	NA	0.982	0.982	0.637	0.406	0.859	0.577	NA
85%	0.966	0.887	0.715	0.637	0.937	0.524	NA	0.948	0.985	0.712	0.568	0.521	0.533	NA
90%	0.959	0.884	0.449	0.784	0.823	0.841	NA	0.936	0.989	0.733	0.745	0.883	0.587	NA
95%	0.970	0.891	0.498	0.804	0.725	0.870	NA	0.977	0.987	0.791	0.910	0.740	0.491	NA
100%	0.985	0.902	0.638	0.794	0.644	0.877	NA	0.975	0.986	0.623	0.670	0.830	0.652	NA
Mean	0.969	0.923	0.643	0.662	0.787	0.724	0.760	0.936	0.985	0.657	0.719	0.748	0.751	0.744
SD	0.012	0.029	0.112	0.098	0.071	0.128	0.099	0.028	0.003	0.090	0.128	0.123	0.146	0.065

(Note: TA, tibialis anterior muscle; MG, medial gastrocnemius muscle; sEMG, surface electromyography; sMMG, surface mechanomyography; NA, not available; the green-highlighted cells indicate the existence of significance in ICC values (*p* < 0.05)).

**Table 3:** Comparison of temporal gait parameters of participating stroke survivors between walking without and with AFO at the paretic side (n = 20).

Interventions	Stance Phase (s)	Swing Phase (s)	Stride Time (s)	Stance Phase (%)	Swing Phase (%)
Without AFO	1.36 ± 0.60	0.66 ± 0.44	2.02 ± 0.19	67 ± 6	33 ± 6
With AFO	1.13 ± 0.32	0.65 ± 0.14	1.79 ± 0.44	63 ± 3	37 ± 3
*p* value	<0.001	<0.001	<0.001	0.018	0.018

(Note: s: second; data are presented as mean ± standard deviation).

## Data Availability

Data is contained within the article.

## References

[1] Laufer Y., Hausdorff J.M., Ring H. (2009). Effects of a foot drop neuroprosthesis on functional abilities, social participation, and gait velocity. Am. J. Phys. Med. Rehab..

[2] Titus A.W., Hillier S., Louw Q.A., Inglis-Jassiem G. (2018). An analysis of trunk kinematics and gait parameters in people with stroke. Afr. J. Disabil..

[3] Kunkel D., Fitton C., Burnett M., Ashburn A. (2014). Physical inactivity post-stroke: A 3-year longitudinal study. Disabil. Rehabil..

[4] Lawan M.M., Lawal I.U., Yusuf A.M. (2022). Correlates of participation restrictions and quality of life among Hausa women with post-stroke disabilities. Bull. Fac. Phys. Ther..

[5] Graham J. (2010). Foot drop: Explaining the causes, characteristics and treatment. Br. J. Neurosci. Nurs..

[6] Kang G.E., Frederick R., Nunley B., Lavery L., Dhaher Y., Najafi B., Cogan S. (2021). The Effect of Implanted Functional Electrical Stimulation on Gait Performance in Stroke Survivors: A Systematic Review. Sensors.

[7] Mijic M., Schoser B., Young P. (2022). Efficacy of functional electrical stimulation in rehabilitating patients with foot drop symptoms after stroke and its correlation with somatosensory evoked potentials—A crossover randomised controlled trial. Neurol. Sci..

[8] Daryabor A., Arazpour M., Aminian G. (2018). Effect of different designs of ankle-foot orthoses on gait in patients with stroke: A systematic review. Gait Posture.

[9] Everaert D.G., Stein R.B., Abrams G.M., Dromerick A.W., Francisco G.E., Hafner B.J., Huskey T.N., Munin M.C., Nolan K.J., Kufta C.V. (2013). Effect of a Foot-Drop Stimulator and Ankle–Foot Orthosis on walking performance after stroke. Neurorehab. Neural. Re..

[10] Yamamoto M., Shimatani K., Hasegawa M., Murata T., Kurita Y. (2019). Effects of altering plantar flexion resistance of an ankle-foot orthosis on muscle force and kinematics during gait training. J. Electromyogr. Kines..

[11] Murayama M., Yamamoto S. (2020). Gait and muscle activity changes in patients in the recovery phase of stroke with continuous use of Ankle–Foot Orthosis with plantarflexion resistance. Prog. Rehabil. Med..

[12] Hesse S., Werner C., Matthias K., Stephen K., Berteanu M. (1999). Non-velocity-related effects of a rigid double-stopped ankle–foot orthosis on gait and lower limb muscle activity of hemiparetic subjects with an equinovarus deformity. Stroke.

[13] Lairamore C., Garrison M.K., Bandy W., Zabel R. (2011). Comparison of tibialis anterior muscle electromyography, ankle angle, and velocity when individuals post stroke walk with different orthoses. Prosthet. Orthot. Int..

[14] Ohata K., Yasui T., Tsuboyama T., Ichihashi N. (2011). Effects of an ankle-foot orthosis with oil damper on muscle activity in adults after stroke. Gait Posture.

[15] Mulroy S.J., Eberly V.J., Gronely J.K., Weiss W., Newsam C.J. (2010). Effect of AFO design on walking after stroke: Impact of ankle plantar flexion contracture. Prosthet Orthot Int..

[16] Nikamp C., Buurke J., Schaake L., Palen J., Rietman J., Hermens H. (2019). Effect of long-term use of ankle-foot orthoses on tibialis anterior muscle electromyography in patients with sub-acute stroke: A randomized controlled trial. J. Rehabil. Med..

[17] Geboers J.F., Drost M.R., Spaans F., Kuipers H., Seelen H.A. (2002). Immediate and long-term effects of ankle-foot orthosis on muscle activity during walking: A randomized study of patients with unilateral foot drop. Arch. Phys. Med. Rehabil..

[18] Banks C.L., Huang H.J., Little V.L., Patten C. (2017). Electromyography exposes heterogeneity in muscle co-contraction following stroke. Front. Neurol..

[19] Swank C., Almutairi S., Wang Price S., Gao F. (2020). Immediate kinematic and muscle activity changes after a single robotic exoskeleton walking session post-stroke. Top. Stroke Rehabil..

[20] Lyu P.Z., Zhu R.T.L., Ling Y.T., Wang L.K., Zheng Y.P., Ma C.Z.H. (2022). How paretic and non-paretic ankle muscles contract during walking in stroke survivors: New insight using novel wearable ultrasound imaging and sensing technology. Biosensors.

[21] Ramsay J.W., Barrance P.J., Buchanan T.S., Higginson J.S. (2011). Paretic muscle atrophy and non-contractile tissue content in individual muscles of the post-stroke lower extremity. J. Biomech..

[22] Nordez A., Jolivet E., Sudhoff I., Bonneau D., de Guise J.A., Skalli W. (2009). Comparison of methods to assess quadriceps muscle volume using magnetic resonance imaging. J. Magn. Reson. Imaging.

[23] Liu W., Wu H.D., Ling Y.T., Shea Q.T.K., Nazari V., Zheng Y.P., Ma C.Z. (2023). Reliability and validity of assessing lower-limb muscle architecture of patients with cerebral palsy (CP) using ultrasound: A systematic review. J. Clin. Ultrasound.

[24] Monjo H., Fukumoto Y., Asai T., Shuntoh H. (2018). Muscle thickness and echo intensity of the abdominal and lower extremity muscles in stroke survivors. J. Clin. Neurol..

[25] Gonzalez-Buonomo J., Pham A.H., Ghuman J., Malik A., Yozbatiran N., Francisco G.E., Frontera W.R., Li S. (2023). Ultrasound Assessment of Spastic Muscles in Ambulatory Chronic Stroke Survivors Reveals Function-Dependent Changes. J. Rehabil. Med..

[26] Roots J., Trajano G.S., Fontanarosa D. (2022). Ultrasound elastography in the assessment of post-stroke muscle stiffness: A systematic review. Insights Into Imaging.

[27] Ma C.Z., Ling Y.T., Shea Q.T.K., Wang L.K., Wang X.Y., Zheng Y.P. (2019). Towards wearable comprehensive capture and analysis of skeletal muscle activity during human locomotion. Sensors.

[28] Hoffman H. (2022). The Brunnstrom Stages of Stroke Recovery. https://www.saebo.com/blog/the-stages-of-stroke-recovery/.

[29] Aboubakr S., Abu-Ghosh A., Ackley W.B., Adolphe T.S., Aeby T.C., Aeddula N.R., Agadi S., Agasthi P., Harb A., Kishner S. (2023). StatPearls.

[30] Downs S. (2015). The Berg Balance Scale. J. Physiother..

[31] Blum L., Korner-Bitensky N. (2008). Usefulness of the Berg Balance Scale in stroke rehabilitation: A systematic review. Phys. Ther..

[32] Rancho Los Amigos National Rehabilitation Center (2001). Observational Gait Analysis.

[33] Ling Y.T., Ma C.Z.-H., Shea Q.T.K., Zheng Y.-P. (2020). Sonomechanomyography (SMMG): Mapping of skeletal muscle motion onset during contraction using ultrafast ultrasound imaging and multiple motion sensors. Sensors.

[34] Choo H., Kwong E., Shek P.T.-C., Leung M.-T., Zheng Y.-P., Lam W.Y.S. (2022). Temporal measures of oropharyngeal swallowing events identified using ultrasound imaging in healthy young adults. PLoS ONE.

[35] Prenton S., Hollands K., Kenney L., Onmanee P. (2018). Functional electrical stimulation and ankle foot orthoses provide equivalent therapeutic effects on foot drop: A meta-analysis providing direction for future research. J. Rehabil. Med..

[36] Ma C.Z.-H., Zheng Y.-P., Lee W.C.-C. (2018). Changes in gait and plantar foot loading upon using vibrotactile wearable biofeedback system in patients with stroke. Top. Stroke Rehabil..

